# Exploring the Femtosecond Filamentation Threshold in Liquid Media Using a Mach–Zehnder Interferometer

**DOI:** 10.3390/s23229163

**Published:** 2023-11-14

**Authors:** Yun Zhang, Yu Xia, Canneng Liang, Anmin Chen, Suyu Li, Mingxing Jin

**Affiliations:** 1 Institute of Atomic and Molecular Physics, Jilin University, Changchun 130012, China; zhangyun18@mails.jlu.edu.cn (Y.Z.); xiayu22@mails.jlu.edu.cn (Y.X.); liangcn22@mails.jlu.edu.cn (C.L.); amchen@jlu.edu.cn (A.C.); mxjin@jlu.edu.cn (M.J.); 2 Research Center for Intelligent Transportation, Zhejiang Laboratory, Hangzhou 311121, China

**Keywords:** femtosecond filament, filamentation threshold, supercontinuum, interference

## Abstract

We experimentally studied the supercontinuum induced by femtosecond filamentation in different liquid media. Using a Mach–Zehnder interferometer, we determined the relative filamentation thresholds (*P*_th_) of these media. Research has shown that the value of the filamentation threshold is greater than that of *P*_cr_ (critical power for self-focusing), which can mainly be attributed to the strong dispersion effect. Changing the focal length of the focusing lens affects filamentation dynamics, thereby affecting the measured results regarding the filamentation threshold. With shorter focal lengths, the linear focusing (i.e., geometrical focusing) regime dominates, and the measured values of *P*_th_ for different liquid media are almost the same; as the focal length becomes larger, self-focusing starts to play a role, making the values of *P*_th_ for different media different from each other. This study presents an efficient method for investigating the femtosecond filamentation phenomenon in liquid media, helpful to provide further insights into the physical mechanism of supercontinuum generation via femtosecond filamentation in liquid media.

## 1. Introduction

Since the initial discovery of laser filamentation in air by Braun et al. [1], extensive theoretical and experimental research has been conducted on femtosecond laser filamentation in transparent media [2,3,4,5]. This nonlinear phenomenon arises from the interaction between Kerr self-focusing and plasma defocusing effects [2]. If the peak power of the femtosecond laser pulse is higher than the critical power of self-focusing *P*_cr_, the phenomenon of self-focusing will occur when the laser pulse propagates in a transparent medium. When the pulse intensity exceeds the ionization threshold of the medium, molecules are ionized, resulting in the generation of numerous plasmas. Then, the defocusing effect of the plasmas balances the self-focusing effect, thereby constraining the peak intensity of the pulse. The long and bright plasma channel formed at this time is called a “filament”. The peak power at which the femtosecond filament is initially formed is referred to as the filamentation threshold *P*_th_. When the peak power exceeds *P*_th_, femtosecond filamentation occurs, accompanied by various nonlinear phenomena such as fluorescence radiation [6], colored conical emission [7], and supercontinuum (SC) generation. Once these phenomena are detected experimentally, it means that femtosecond filamentation has occurred. Over the past few decades, numerous theoretical and experimental studies have been carried out to explore the SC generation induced by femtosecond filamentation [8,9].

Femtosecond laser pulses can generate the SC in transparent media, spanning a broad spectral range from visible wavelengths to near-infrared. The spectral range of the SC is determined by key factors such as self-phase modulation (SPM), dispersion, intensity clamping, self-steepening, and other nonlinear processes [10,11,12,13,14]. A femtosecond filament can be formed in a variety of transparent media [15], and the spectral shape of the SC induced by the filaments is similar, indicating that the generation of the SC is a common feature of the interplay between ultrashort laser pulses and the substance. Since the SC maintains the polarization characteristics and spatiotemporal coherence of the original laser pulse over its entire spectral range, it is widely used as a broadband light source in time-resolved spectrum [16,17], optical communication [18], remote sensing [19,20], biomedical imaging [21], few-cycle femtosecond pulse generation [22,23], etc.

Compared with gaseous media, condensed state media (solids and liquids included) have higher nonlinear refractivity, making it is easier to generate filaments as well as SC with relatively high intensity. For solid media, they may suffer from optical damage under the action of intense laser pulses. In contrast, liquid media can easily change their thickness, making them attractive for many experiments in nonlinear optics. There are numerous reports about SC generation in liquid media [24,25,26]. For example, Wittmann et al. [27] studied how femtosecond pulses undergo spectral broadening when tightly focused in different media, including fused silica, ethanol, water, and heavy water. In addition, research has been conducted to regulate the production of the SC in transparent liquid media, such as adding a small amount of protein to the water to inhibit spectral broadening [28,29] or doping the water with metal nanoparticles to enhance spectral broadening [30].

The *P*_cr_ and *P*_th_ of femtosecond lasers in transparent media have been studied extensively [2,31,32]. Typically, for pulses with a long pulse duration, such as a picosecond pulse, *P*_th_ is almost identical to *P*_cr_. This similarity is also true in a lower dispersion medium, such as air. However, for higher dispersion media or ultrashort pulses, *P*_th_ is higher than *P*_cr_. As for the measurement of *P*_th_ and *P*_cr_, quite a few methods have been developed. For example, by using the moving focus method, the value of *P*_cr_ under air [33], helium [34], and flaming conditions [35] has been measured. Liang et al. [36] used a photomultiplier tube to measure fluorescence and experimentally determine the value of *P*_cr_ for femtosecond vortex beams in atmospheric conditions. Akturk et al. [37] proposed a P-scan method to successfully distinguish moving focus, linear, filamentation, and multi-filamentation regimes in gases and obtained the *P*_cr_ associated with a femtosecond Gaussian beam.

Since the direct observation of femtosecond filaments in liquid media is not convenient, it is necessary to develop new approaches to diagnose filaments in experiments. By detecting the typical nonlinear phenomena accompanying femtosecond filamentation, information about femtosecond filamentation can be deduced. Based on this principle, we used a Mach–Zehnder interferometer (MZI) in our previous work to investigate the interference of SC and determined the actual value of *P*_th_ in the water [38]. The SC that we regard as a coherent light source is induced by femtosecond filamentation and the spectral signal passing through the MZI, resulting in the formation of interference patterns. For this reason, it is reasonable to judge the initiation of femtosecond filamentation based on the emergence of interference patterns in the field of view.

For this paper, the SC induced by femtosecond filaments and the *P*_th_ in different liquid media were measured. By using the MZI, we measured the relative measurement values for the filamentation threshold *P*_th_ for higher dispersion media, and in this paper, we attempt to analyze the discrepancy between the measured values of *P*_th_ and theoretical value of *P*_cr_. In addition, the influence of the focal length of the focusing lens on the relative measurement value of *P*_th_ is also discussed.

## 2. Experimental Setup

Considering that this study is a more in-depth study based on the work described in Ref. [38], the same experimental device deployed for that study was adopted for this one. See Ref. [38] for a diagram of the experimental device. The experiment was conducted using a femtosecond laser system under atmospheric conditions (Solstice Ace, Spectra-Physics, 1565 Barber Lane, Milpitas, CA, USA). The pulse duration was 35 fs, with a central wavelength of 800 nm and a repetition rate of 1 kHz. For the experiment, five different liquid sample were selected: water, anhydrous ethanol, 95% ethanol, NaCl solution, and glucose solution. The concentrations of the NaCl and glucose solutions were 100 mg/mL, 50 mg/mL, and 25 mg/mL, respectively. Femtosecond laser pulses were focused by lenses with focal lengths of 1000 mm, 750 mm, 400 mm, and 250 mm to form filaments in different solutions. Each time the focusing lens is replaced, the position of the collimating lens needs to be adjusted to re-collimate the SC. An interference pattern is generated after the SC passes through the MZI. When the CCD can capture the interference pattern, the corresponding pulse energy can be measured using a pulse energy meter.

## 3. Results and Discussion

### 3.1. Supercontinuum Generation

**Figure 1:**
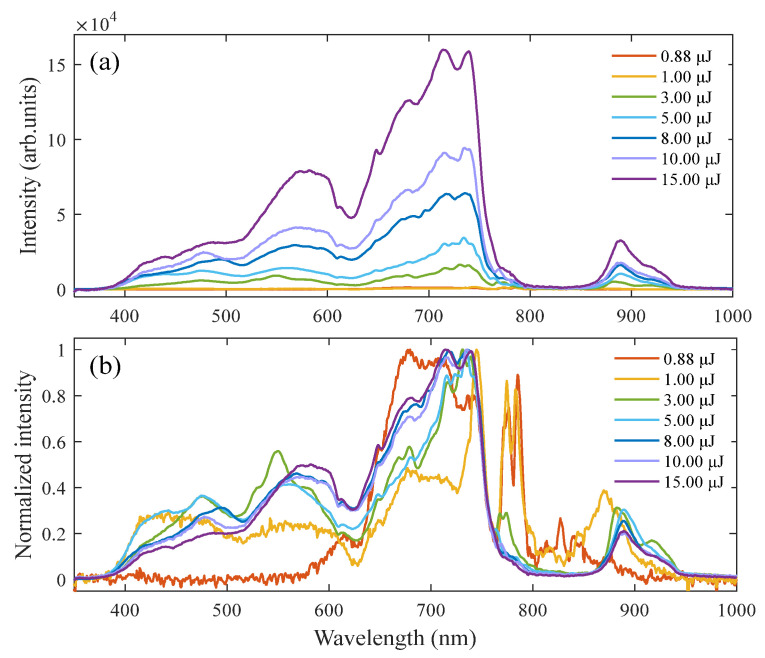
(**a**) SC produced when different incident pulse energies form filaments in water; (**b**) the normalized spectra of (**a**).

When femtosecond laser pulses are transmitted through transparent medium and form filaments, nonlinear phenomena like the self-steepening and SPM result in spectral broadening, which covers the entire visible light band and is also known as supercontinuum/white light [39,40]. Figure 1 shows the SC produced by a laser with different incident pulse energies forming a filament in water through a focusing lens with a focal length of 400 mm. In liquid media, it is difficult to observe the femtosecond filament directly. The generation of the SC can usually be regarded as a sign of filament formation [2,41].

As can be seen from Figure 1a, the SC is generated and the spectral range encompasses the entire visible wavelength range when the pulse energy reaches around 0.88 μJ. In addition, the spectral intensity of the SC increases as the incident pulse energy increases. From Figure 1b, it is obvious that the normalized spectra undergo asymmetric broadening, with a stronger shift towards the blue side than the red side. This asymmetric broadening is caused by various processes, including self-steepening, space-time focusing, and plasma formation resulting from the creation of free electrons generated by multiphoton ionization (MPI) [28,42]. As the incident pulse energy increases, the signal intensity ratio between the blue side component and the center wavelength of the spectrum increases rapidly. It can also be observed that the spectrum extends to around 380 nm, and this value remains consistent regardless of the incident pulse energy. In other words, spectral broadening is not significantly influenced by the change in pulse energy/power, and intensity clamping is an important factor limiting the SC. Furthermore, linear chromatic dispersion [43], multi-photon absorption, and plasma defocusing have significant influences on SC suppression [13]. It is necessary to consider the differences between the SC generated in different liquid media.

**Figure 2:**
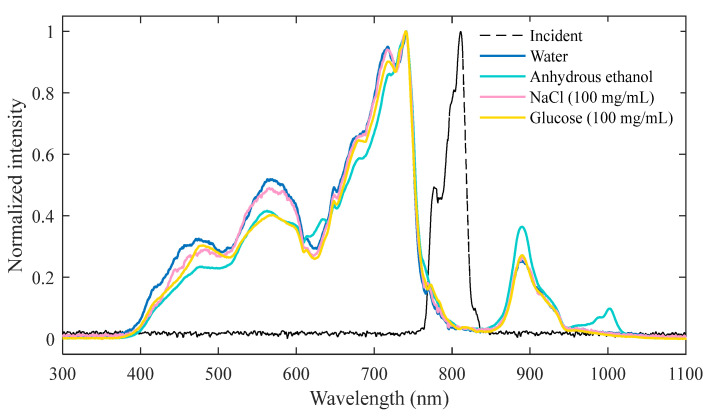
SC generated by using a focusing lens with *f* = 400 mm in different liquid media when the incident pulse energy is 10 μJ.

**Figure 3:**
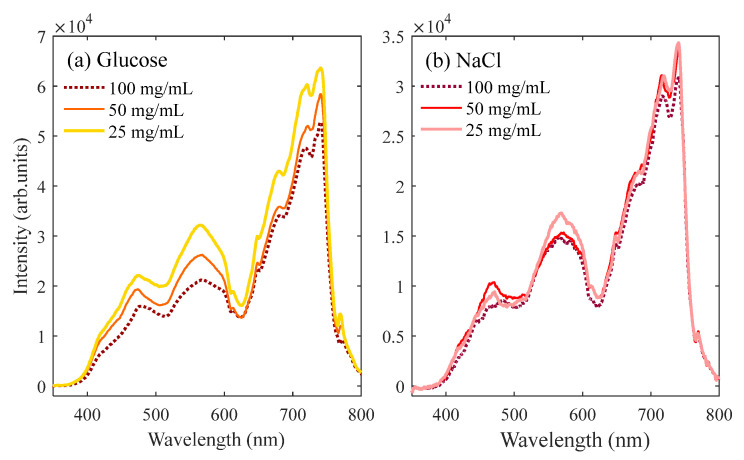
SC generated in (**a**) glucose and (**b**) NaCl solutions with different concentrations using a 400 mm focusing lens.

In Figure 2, a distinct dip near 620 nm can be seen in the SC, which is attributed to the inverse Raman effect induced by water molecules [29,44]. The same dip in SC generated in anhydrous ethanol is due to the ionization of anhydrous ethanol, which produces water molecules. Therefore, the position of the dip remains consistent regardless of factors such as variations in laser intensity, the quantity of additives in the water, and focusing conditions, as described in Ref. [29]. From Figure 2, it can be observed that the inclusion of additives suppresses the blue portion of the white light spectrum in comparison to pure water. At the same pulse energy, the intensity of the SC signals produced in different media varies, while the spectral shape and spectral broadening range almost remain consistent. In addition, we explored the SC generated in glucose and NaCl solutions when the pulse energy is 10 μJ, as shown in Figure 3. 

Figure 3a shows the SC generated in different concentrations of glucose solutions. Obviously, with the increase in glucose concentration, the suppression of the blue components in the spectrum becomes more pronounced. Figure 3b shows the SC generated in the NaCl solutions, and the results are similar to those in the glucose solutions. The blue-side of the SC is significantly suppressed, indicating that the addition of additives can affect the process of free electron generation resulting from the MPI. The difference in electron density will inevitably affect the self-phase modulation and affect the generation of white light. The electron density decreases with an increase in the additives in water; thus, the plasma effect is weakened, resulting in the suppression of the spectrum in the short-wavelength direction. In other words, the additives act as an electron capture in water. Moreover, the absorption and scattering of light by additives in water may also contribute to the suppression of the SC [45].

### 3.2. Self-Focusing Critical Power and Filamentation Threshold

**Figure 4:**
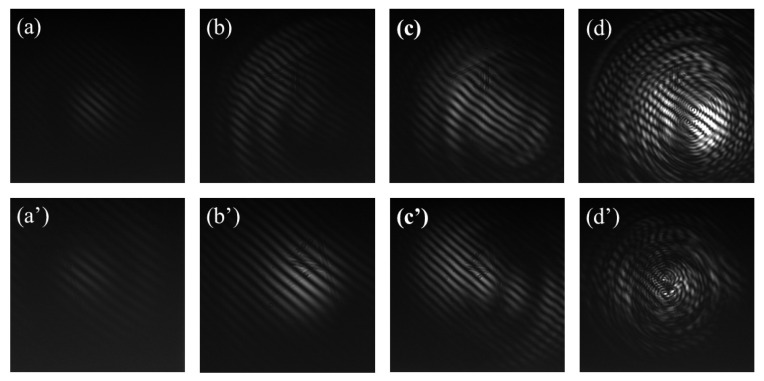
Interference patterns in water with pulse energies of (**a**) 0.88 μJ, (**b**) 1.0 μJ, (**c**) 2.0 μJ, and (**d**) 4.0 μJ, respectively. (**a’**–**d’**) Interference patterns in anhydrous ethanol at the corresponding pulse energies. The experiments were performed using a focusing lens with *f* = 400 mm.

Femtosecond filamentation is usually accompanied by SC generation; hence, the presence of the SC suggests that a femtosecond filament has been formed. It seems that by measuring the SC spectrum, one can determine the onset of femtosecond filamentation (i.e., the filamentation threshold *P*_th_). Under low pulse energy conditions, the spectrometer may be unable to detect certain spectral components due to limitations in its detection efficiency; thus, the actual value of *P*_th_ is difficult to determine using spectroscopic technology. To overcome this problem, interferometry is adopted, which can improve detection sensitivity. In our experiment, the interference patterns were captured using a CCD. The pulse power at which the interference pattern starts to emerge can be defined as the actual value of *P*_th_. We captured the interference patterns generated by the 600 nm spectral signal in pure water and anhydrous ethanol, as shown in Figure 4.

If the pulse energy is insufficient to produce a SC, the interference pattern cannot be captured by the CCD. However, when the pulse energy exceeds a certain value, for example, 0.88 μJ, as illustrated in Figure 4a, flickering interference patterns can be observed in the field of view, indicating the occurrence of femtosecond filamentation in the water. A similar filamentation effect can be observed in anhydrous ethanol when the incident pulse energy is approximately 0.88 μJ, as shown in Figure 4a’. This pulse power can be defined as the filamentation threshold *P*_th_, and in our previous work [24,38], we defined the filamentation threshold *P*_th_ using this approach. As the pulse energy increases to 1.0 μJ, a stable interference pattern with clear fringes is observed. By further increasing the pulse energy to from multiple filaments, more complex interference patterns are generated, and the interference pattern eventually becomes undistinguishable, as shown in Figure 4b–d. Using the MZI, the actual filamentation threshold in liquid media at a low pulse energy can be accurately determined via interferometry. This method is different from conventional methods such as the moving focus method [34,35] and the ablation method [46], which are typically used for determining *P*_cr_ or *P*_th_. Both of these methods have their respective limitations; the former requires the information of the focus position, and the latter is not applicable to situations involving liquid media. In contrast, for interferometry using the MZI system, the filamentation threshold in liquid media is obtained by recording the interference patterns of the SC, which is a more intuitive and convenient method.

If the input pulse is temporally Gaussian-type, the value of *P*_cr_ can be calculated using the following equation:

(1) $$P_{cr}=3.77\lambda_{0}^{2}/8\pi n_{0}n_{2},$$

where $n_{0}$ and $n_{2}$ are the linear and nonlinear refractive indices of the medium [47,48]; $\lambda_{0}^{}$ is the central wavelength of femtosecond laser pulse. The values of $n_{0}$ and $n_{2}$ for water and anhydrous ethanol were taken from Ref. [49]. Based on their values, the values of $n_{0}$ and $n_{2}$ for the 95% ethanol can be estimated. The formula to calculate the refractive index of a mixture is called the “Lorentz-Lorenz equation”, which is given by the following [50]:

(2) $$n_{ab}=\phi_{a}n_{a}+\phi_{b}n_{b},$$

**Figure 5:**
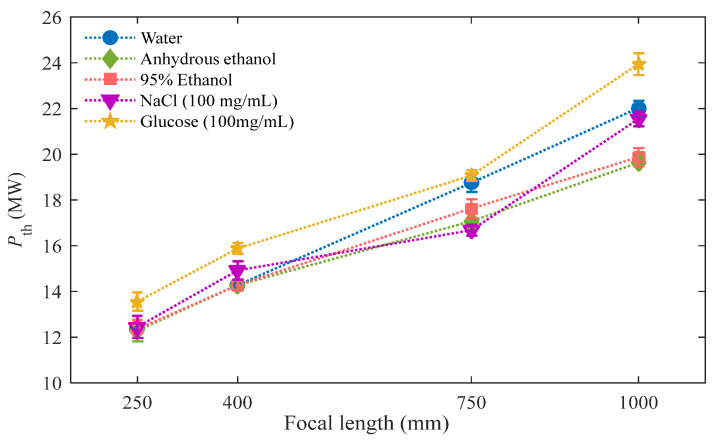
Variations in the filamentation threshold *P*_th_ in different liquid media and with different focal lengths.

$n_{ab}$, $n_{a}$, and $n_{b}$ are the refractive indices of substance 1, substance 2, and the mixture, respectively. $\phi_{a}$ and $\phi_{b}$ are the volume fractions of each component in the solution. Taking the linear refractive index $n_{0}$ as an example, it can be calculated through the values of $n_{0}$ for water ($n_{a}$ = 1.33) and anhydrous ethanol ($n_{b}$ = 1.36) and the respective volume fraction of the mixture ($\phi_{a}$ = 0.05, $\phi_{b}$ = 0.95). Substituting these values into Equation (2), the value of $n_{0}$ for the 95% ethanol ($n_{ab}$) is 1.3585. Thus, it can be concluded that the $n_{2}$ of 95% ethanol is 7.52 × 10^−16^ cm^2^/W. The values of $n_{0}$ and $n_{2}$ for the NaCl (100 mg/mL) solution can calculated using the approach described by Adair et al. [51]. The values of $n_{0}$ for the glucose (100 mg/mL) solution refer to data from Yeh et al. [52]. The following equation can be used to calculate the power (femtosecond filamentation threshold *P*_th_):

(3) $$P=E_{in}/(\sqrt{\pi /2}\tau_{p}),$$

where $\tau_{p}$ is the pulse duration. During the experiment, the femtosecond pulses pass through multiple optical elements, resulting in a broadening of the pulse duration. In our experiment, the pulse duration, measured by an auto-correlator meter placed in front of the focusing lens L_1_, was approximately 50 fs. Limited by the detecting precision of energy meter and due to the fluctuation in the energy of the incident femtosecond pulses, the measured value of the pulse energy fluctuates rather than remaining constant. However, this fluctuation is very small and does not affect the judgment of the filamentation threshold. The *P*_th_ calculated according to the measured value of the incident pulse energy also has a corresponding fluctuation range, which is shown in the form of an error bar in Figure 5. The variations in the value of *P*_th_ with the different focal lengths in different liquid media are shown in Figure 5. Table 1 exhibits the detailed values of *P*_th_ and *P*_cr_ of different media when using focusing lenses with different focal lengths. Some data are missing from Table 1 due to the lack of publications in the literature on the nonlinear refractive index values of glucose solutions.

Figure 5 clearly demonstrates that when using a longer focusing lens (*f* = 1000 mm), there is a significant difference in *P*_th_ in the five kinds of media. As the focal length decreases, *P*_th_ gradually tends to converge to the same values, and they seem to be close to each other when the focal length is *f* = 250 mm. For water, the value of *P*_cr_ is 1.76 MW. In the case of *f* = 250 mm, the value of *P*_th_ is 12.45 MW, while when *f* = 1000 mm, *P*_th_ is 22.02 MW. As the focal length is increased, the value of *P*_th_ gradually increases and always remains higher than that of *P*_cr_. The difference between *P*_th_ and *P*_cr_ can be attributed to the strong dispersion effect. Group velocity dispersion in transparent media increases the pulse duration during propagation and consequently decreases the peak power. The values of *P*_th_ shown in Table 1 and Figure 5 were calculated by using the initial pulse duration, which inevitably makes them much higher than the values of *P*_cr_ [53]. In this work, the generation of the SC serves as a criterion for determining *P*_th_, which means that the filament has been formed, meaning that the value of *P*_th_ obtained via this method is higher than that of *P*_cr_. The high dispersion coefficient of the liquid medium has a significant impact on the filamentation process when the femtosecond laser pulses propagate through it. In short pulses and high dispersion media, dispersion can also play the same role as the plasma defocusing process to delay the focus of the beam. At this time, higher power is required to balance the effect of dispersion [54]. In addition, the dispersion of the focusing lens itself has some effects. When using the same focusing lens, the influence of the dispersion caused by the focusing lens on the measured value of *P*_th_ is consistent and can be ignored. 

The filament core during femtosecond filamentation is supplied with energy from the surrounding energy reservoir, which contains up to 90% of the total pulse energy [55,56,57]. When an external focusing lens is used, the energy reservoir that surrounds the core of the filament is concentrated into a smaller volume, leading to a higher intensity when the laser pulse undergoes self-focusing [58]. A filament is formed when a dynamical balance is achieved between the geometrical focusing, Kerr self-focusing, and defocusing effects resulting from plasma, diffraction, and dispersion. The focal length significantly influences the effective peak plasma density within the plasma column [58]. As a result, changes in the focusing lens affect the dynamics of femtosecond filamentation, and different effects dominate the femtosecond filamentation when the focal length changes. In the case of *f* = 250 mm, the measured values of *P*_th_ for different media are almost the same (12.40 ± 0.30 MW). This is because when *f* = 250 mm, the focal length is significantly shorter than the collapse distance resulting from the femtosecond beam self-focusing, which can be calculated using the semiempirical formula [59,60]; thus, the beam focusing is mainly affected by the focusing lens, that is, linear focusing plays a dominant role in femtosecond filamentation. In the linear focusing regime, an increase in pulse energy leads to an increase in both peak electron density and the diameter of the plasma channel [61]. In the case of tight focusing, the interaction between self-focusing and plasma defocusing does not exert a substantial influence on intensity clamping [62]. It is well known that different liquid media have different refractive indices and dispersion characteristics, which play a certain role in the linear focusing. However, the measured values of *P*_th_ for different liquids are almost consistent when *f* = 250 mm, indicating that the effect of the parameters of the medium on the linear focusing is not significant in tight focusing. Since liquid samples with similar linear refractive indices were used in the experiment, linear refraction had a relatively little effect on the geometrical focusing of the femtosecond beam after passing through the liquid samples. Therefore, with shorter focal lengths, the measured values of *P*_th_ are less affected by the type and concentration of media.

However, as the focal length is increased, the measured values of *P*_th_ increase and the *P*_th_ values for different media vary from each other, as shown in Figure 5 and Table 1. This can be attributed to the fact that the longer the focal length, the weaker the converging ability of the focusing lens is, thus more energy is required to reach the filamentation threshold. As the focal length is increased, the self-focusing effects gradually begin to take effect. The linear and nonlinear refractive indices of liquid media play a greater role in the nonlinear focusing of the light beam. The temporal dispersion caused by a longer focal length lens is so small that we can ignore its effect on the filamentation threshold measurements. Therefore, we can speculate that, with longer focal lengths, self-focusing will play a dominant role, competing with dispersion and photo-ionization, etc., and this depends on the type and concentration of media. Consequently, the differences between the measured values of *P*_th_ for different kind of liquid media will be considerable.

## 4. Conclusions

In this work, the SC generated by femtosecond filamentation in different liquid media has been studied experimentally. It was found that at the same pulse energy, the intensity of the SC signals produced in different media is different, while the spectral shape and spectral broadening range remain almost consistent. The relative measurement value of filamentation threshold *P*_th_ in liquid media can be measured via interferometry using a MZI. Due to the dispersion effect, the value of *P*_th_ is greater than that of *P*_cr_. Additionally, changing the focal length of the focusing lens can affect the filamentation dynamics and therefore affect the measured value of *P*_th_. Under shorter focal lengths, linear focusing plays a major role and the measured values of *P*_th_ for different media are almost the same. This is because the medium parameters have an insignificant effect on linear focusing in tight focusing. However, as the focal length is increased, the value of *P*_th_ increases gradually and the difference between the values of *P*_th_ for different media becomes more obvious. This is because, with longer focal lengths, self-focusing plays a dominant role, and it is more susceptible to the influence of the medium. This study presents an efficient method for investigating the femtosecond filamentation process in liquid media. However, the method also has limitations; for example, the detection efficiency of the CCD used in our experiment was not that high. A CCD with higher detection efficiency can capture clearer patterns. Nevertheless, the relative measurement of *P*_th_ in liquid media provided by this method can still serve as a reference.

## Figures and Tables

**Table 1:** Filamentation threshold (*P*_th_ and *P*_cr_) values derived from using different media and different focal lengths.

Material	$n_{0}$	$n_{2}$	*P*_cr_(MW)	*P*_th_(MW)			
				*f* = 1000 mm	*f* = 750 mm	*f* = 400 mm	*f* = 250 mm
Water	1.33 ^a^	4.1 × 10^−16 a^	1.761	22.02	18.67	14.20	12.45
Anhydrous ethanol	1.36 ^a^	7.7 × 10^−16 a^	0.917	19.63	17.07	14.36	12.29
95% Ethanol	1.3585	7.52 × 10^−16^	0.940	19.79	17.55	14.20	12.29
NaCl (100 mg/mL)	1.3501	4.125 × 10^−16^	1.725	21.54	16.76	14.84	12.45
Glucose (100 mg/mL)	1.3446 ^b^	/	/	23.94	19.15	15.79	13.56

^a^ Reference [49]. ^b^ Reference [52].

## Data Availability

The data presented in this study are available from the corresponding author upon reasonable request.
